# Metabolic, Apoptotic and Fibro-Inflammatory Profiles of the Heart Exposed to Environmental Electromagnetic Fields

**DOI:** 10.3390/ijms241411709

**Published:** 2023-07-20

**Authors:** Lesia Savchenko, Ilenia Martinelli, Dimitri Marsal, Oksana Batkivska, Vyacheslav Zhdan, Igor Kaidashev, Nathalie Pizzinat, Frederic Boal, Helene Tronchere, Junwu Tao, Oksana Kunduzova

**Affiliations:** 1 National Institute of Health and Medical Research (INSERM) U1297, CEDEX 4, 31432 Toulouse, France; lesia.savchenko@inserm.fr (L.S.); ilenia.martinelli@unicam.it (I.M.); dimitri.marsal@inserm.fr (D.M.); oksana.batkivska@inserm.fr (O.B.); nathalie.pizzinat@inserm.fr (N.P.); frederic.boal@inserm.fr (F.B.); helene.tronchere@inserm.fr (H.T.); 2 University Toulouse III, 118 Route de Narbonne, CEDEX 9, 31062 Toulouse, France; tao@laplace.univ-tlse.fr; 3 Poltava State Medical University, 23 Shevchenko, 36000 Poltava, Ukraine; fmedicine1997@gmail.com (V.Z.); kaydashevip@gmail.com (I.K.); 4 Department of Functional and Laboratory Diagnostics, I. Horbachevsky Ternopil National Medical University, 1 Maidan Voli, 46001 Ternopil, Ukraine; 5 LAPLACE, INP-ENSEEIHT, 2 Rue Camichel, 31071 Toulouse, France

**Keywords:** electromagnetic stressor, apoptosis, inflammation, fibrosis, antioxidants

## Abstract

Environmental stress can disturb the integrative functioning of the cardiovascular system and trigger a number of adaptive and/or maladaptive cell responses. Concomitant with the expanding use of mobile communication systems, public exposure to electromagnetic fields (EMFs) raises the question of the impact of 900 MHz EMFs on cardiovascular health. Therefore, in this study, we experimentally investigated whether 915 MHz EMF exposure influenced cardiac metabolic, antioxidant, apoptotic, and fibro-inflammatory profiles in a mouse model. Healthy mice were sham-exposed or exposed to EMF for 14 days. Western blot analysis using whole cardiac tissue lysates demonstrated that there was no significant change in the expression of oxidative phosphorylation (OXPHOS) complexes between the control and EMF-exposed mice. In addition, the myocardial expression of fibro-inflammatory cytokines, antioxidant enzymes, and apoptosis-related markers remained unchanged in the EMF-challenged hearts. Finally, the structural integrity of the cardiac tissues was preserved among the groups. These findings suggest that the apoptotic, antioxidant, metabolic, and fibro-inflammatory profiles of the heart remained stable under conditions of EMF exposure in the analyzed mice.

## 1. Introduction

Exposure to electromagnetic waves in the human environment is continuously increasing and currently reaching uncontrolled levels. Electromagnetic fields (EMFs) are generated from natural environments and manmade sources and affect biological systems [1,2,3]. The cardiovascular system is a main concern with regard to the impact of EMF since mobile phone use involves everyday exposure to EMFs and can predispose users to cell, tissue, and organ dysfunctions [4,5,6]. The majority of studies in this area have focused on neurological disease [6,7], cancer [8,9], reproductive disorders [10,11], immune dysfunction [7,12,13,14], and cognitive effects [15,16]. However, the biological effects of EMF exposure on cardiovascular health remain unclear because of the conflicting findings of various studies conducted using animal models and humans [14,17,18]. Studies conducted with rats subjected to electromagnetic stress showed that cardiac apoptosis, oxidative stress, and morphologic abnormalities can be triggered by long-term exposure to EMFs [19,20]. However, another two studies reported that there was no influence on the cardiovascular system among rats subjected to chronic exposure to an EMF [21,22]. The heart is a highly coordinated organ with the notable ability to adapt in response to physiological or pathological stresses in order to maintain homeostasis. Defects in cardiac antioxidant defense, metabolism, and the immune system are major research hotspots with respect to the risk factors of cardiovascular complications [23,24,25,26]. Unresolved inflammation can predispose patients to cardiovascular events or affect the prognosis of a failing heart [27,28]. The heart is also responsible for maintaining its own homeostasis to support continuous energetic demands [29,30]. The cardiac tissues require high energy expenditure and must continuously generate large amounts of adenosine triphosphate (ATP) through oxidative phosphorylation (OXPHOS) to sustain contractile function [31,32,33]. This process requires a considerable quantity of oxygen and calibrates the formation of reactive oxygen species (ROS) [34,35]. An imbalance toward the pro-oxidative state induces oxidative stress, cell death, and inflammation [36]. A number of defense mechanisms have evolved to provide a balance between the production and removal of ROS. Superoxide dismutase (SOD) catalyzes the conversion of superoxide radicals into hydrogen peroxide (H_2_O_2_) and molecular oxygen. In the peroxisomes of eukaryotic cells, the enzyme catalase converts H_2_O_2_ to water and oxygen, thereby completing the detoxification initiated by SOD [37,38,39]. Recent studies suggest that EMFs can affect antioxidant enzyme status, signal transduction, and protein and gene expression, which play a decisive role in regulating cellular metabolic functions [4]. However, there is still a gap in the understanding of EMF-generated cardiac cell responses, and the impacts of EMF exposure on the pathogenesis of cardiovascular complications remain unclear. 

The global pervasiveness of mobile phones and internet-connected devices in society raises the question of the impact of 900 MHz EMFs on the heart. Therefore, in this study, we experimentally investigated whether 915 MHz EMF exposure influenced cardiac tissue integrity, metabolic, apoptotic, and fibro-inflammatory profiles in a mouse model.

## 2. Results

**Figure 1:**
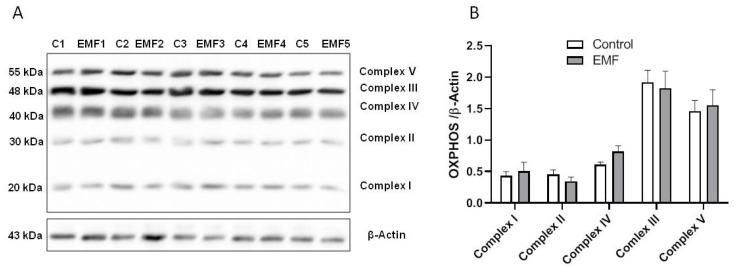
Mitochondrial OXPHOS system in mice subjected to 915 MHz EMF for 14 days. (**A**) Representative Western blot image and (**B**) quantification of MitoProfile Total OXPHOS protein expression levels in control and EMF-exposed mice after 14 days. The results are presented as means ± SEM.

Mitochondrial dysfunction can result from a disruption of the OXPHOS chain, leading to abnormal energy and ROS production [36,40]. We first examined whether electromagnetic stress affects cardiac OXPHOS components in mice subjected to 915 MHz EMFs for 14 days. To detect the OXPHOS complexes simultaneously, the total proteins were extracted from cardiac tissue for immunoblotting using a total OXPHOS rodent antibody cocktail kit (Figure 1A). As shown in Figure 1B, the myocardial expression levels of mitochondrial OXPHOS complexes I to V were not significantly different between the control and EMF-exposed mice.

**Figure 2:**
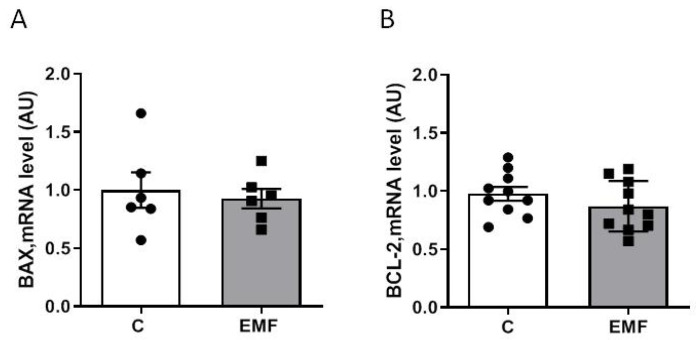
Cardiac mRNA expression of apoptosis-associated genes in mice exposed to EMFs. (**A**,**B**) qRT-PCR quantification of the mRNA-expression levels of *BAX* and *BCL-2* in control and EMF-exposed mice after 14 days. The results are presented as means ± SEM.

Next, we examined whether exposure to EMFs for 14 days induces cardiac tissue apoptosis in mice. Gene expression was assessed using quantitative polymerase chain reaction (qRT-PCR). Apoptotic gene expression was evaluated based on the *BAX* gene (BCL-2 Associated X-protein) and B-cell lymphoma 2 (*BCL-2*). Analysis of apoptosis-associated genes demonstrated that the myocardial expression levels of *BAX* (Figure 2A) and *BCL-2* (Figure 2B) were not significantly different between the control and EMF-challenged groups.

**Figure 3:**
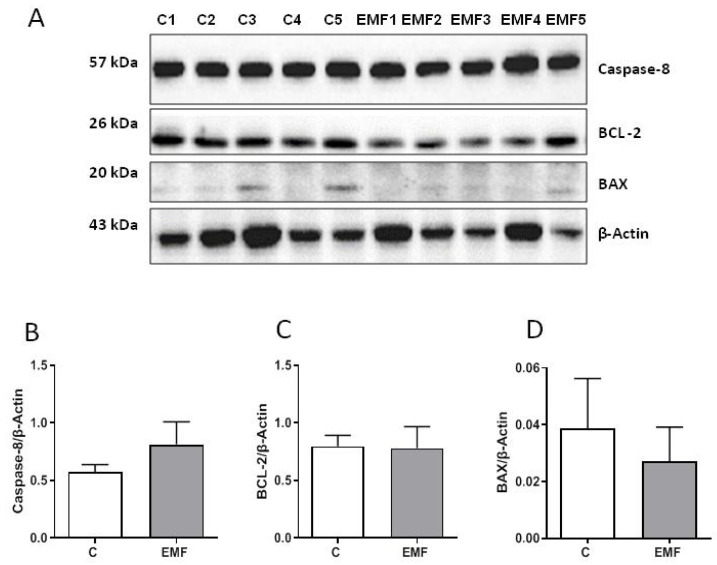
Myocardial levels of apoptosis-related proteins in EMF-challenged hearts. (**A**) Representative Western blot images and (**B**–**D**) quantification of protein expression levels of caspase-8, BCL-2, and BAX in cardiac tissue from mice subjected to 14 days of EMFs. The results are presented as means ± SEM.

Furthermore, protein expression of apoptosis-related factors caspase-8, BAX, and BCL-2 was examined using Western blotting for the control and EMF-exposed mice. As shown in Figure 3A–D, the cardiac expression of caspase-8, BAX, and BCL-2 was stable among the control and EMF-challenged groups.

**Figure 4:**
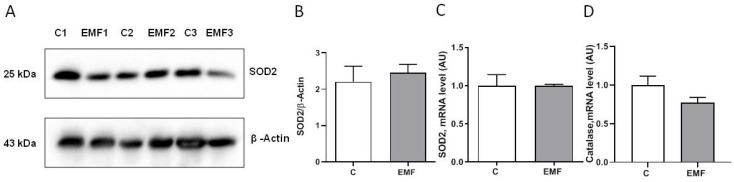
Cardiac expression of SOD2 in EMF-exposed mice. (**A**) Representative Western blot image and (**B**) quantification of SOD2 protein expression level. (**C**,**D**) qRT-PCR quantification of the expression level of *SOD2* and *catalase* in mice exposed to 915 MHz EMFs and control mice after 14 days. The results are presented as means ± SEM.

To examine antioxidant statuses of the EMF-challenged mice, we assessed the cardiac expression of SOD2, a key component of the mitochondrial antioxidant defense and catalase. As shown in Figure 4A–C, the SOD2 protein and mRNA expression levels were unchanged between the control and EMF-exposed mice. *Catalase* mRNA levels did not differ significantly in the hearts exposed to EMFs compared to control group (Figure 4D).

**Figure 5:**
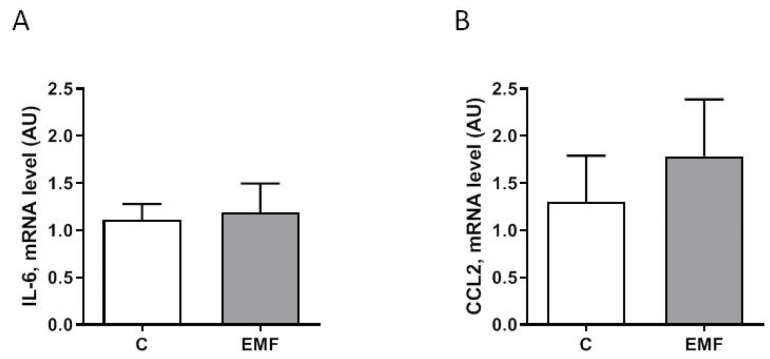
Pro-inflammatory profiling in cardiac tissue from EMF-exposed mice. (**A**,**B**) qRT-PCR results regarding *IL-6* and *CCL2* in control group, and mice subjected to EMFs for 14 days. The results are presented as means ± SEM.

Exposure to electromagnetic stress can modulate inflammatory responses, signal transduction pathways, and downstream protein factors relevant to stressful signals [13,15]. As shown in Figure 5A,B, the myocardial expression of pro-inflammatory factors including interleukin-6 (*IL-6*) and C-C motif chemokine ligand 2 (*CCL2*) was not significantly different between the control and EMF-exposed groups.

**Figure 6:**
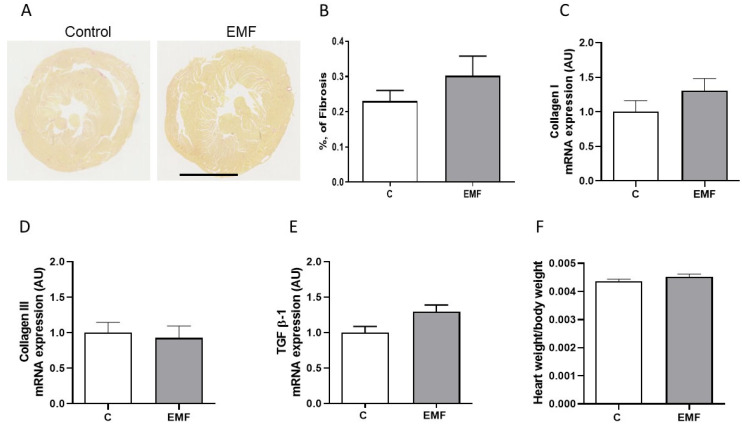
Cardiac tissue integrity in EMF-challenged mouse hearts. (**A**) Representative images showing Sirius red staining of cardiac sections in control and EMF-exposed mice for 14 days (scale bar: 2.5 mm) and (**B**) quantification of (**A**). (**C**–**E**) qRT-PCR analysis of fibrosis-associated genes including *collagen type I*, *collagen type III* and *TGF β-1* and (**F**) ratio of heart weight to body weight in control and EMF-exposed groups. The results are presented as means ± SEM.

To address the effects of EMF exposure on cardiac structural integrity, we next evaluated the cardiac content of collagen, the main fibrous protein in the extracellular matrix (ECM), using picrosirius red staining in the mice subjected to EMFs for 14 days. Analysis of myocardial fibrosis conducted on the control and EMF-exposed mice demonstrated that exposure to a 915 MHz EMF does not affect the collagen content in cardiac tissues (Figure 6A,B).

Investigating the relationships between changes in structural levels and gene expression is crucial; therefore, we examined the cardiac expression of the major constituents of cardiac ECM *collagen types I* and *III* and multifunctional cytokine transforming growth factor beta 1 (*TGF β-1*) in the EMF-challenged hearts. As shown in Figure 6C–E, the myocardial expression levels of *collagen types I* and *III* and *TGF β-1* were not significantly different between the control and EMF-exposed mice. Furthermore, the heart weight adjusted to body weight was analyzed among the mice subjected to EMFs for 14 days. The total heart-weight-to-body-weight ratios were not significantly different among both groups (Figure 6F).

## 3. Discussion

Biological systems are very sensitive to changes in the biological environment and react to the presence of an EMF. The expansion of mobile communication systems operating in the 900 MHz spectrum contributes to the open debate concerning the potential effects of EMFs on human health. The overwhelming majority of such studies focus on the brain since cell phones are held close to the head during use and can alter neural functions in humans [41,42,43]. However, the potential effects of EMFs on cardiovascular health remain largely obscure. This study was conducted to evaluate the effects of 915 MHz EMF exposure on cardiac tissue integrity and metabolic, antioxidant, apoptotic and fibro-inflammatory profiles in mice. We demonstrated that the myocardial expression of fibro-inflammatory genes, antioxidant enzymes, mitochondrial OXPHOS complexes, and apoptosis-related markers remained unchanged in the EMF-exposed mice after 14 days as compared to control animals. Furthermore, the structural integrity of cardiac tissue was preserved in EMF-challenged hearts. These data suggest that tissue structure and metabolic, antioxidant and fibro-inflammatory profiles of the mouse hearts were unaltered by EMF exposure for 14 days. 

The exploration of the biological effects of EMFs on the living system is a complex research area. To date, there have been few scientific reports in which major cardiac remodeling processes that integrate metabolic, apoptotic, antioxidant, and fibro-inflammatory profiles were examined in EMF-exposed living systems in a synchronous manner. The mammalian heart is equipped with mechanisms to adapt to environmental stress, and one of the keys to this efficient response is rooted in alterations that take place in the mitochondrial metabolism. In cardiac cells, mitochondrial OXPHOS complex activities are not only responsible for the generation of high-energy phosphates but are also involved in a variety of cellular processes, including ROS production, apoptosis, and inflammation [44,45]. In the current study, we examined myocardial OXPHOS machinery in conjunction with apoptotic status of the myocardium subjected to electromagnetic stress for 14 days. Our results suggest that the myocardial expression levels of mitochondrial OXPHOS complexes I–V and the apoptosis-related proteins including BAX, BCL-2, and caspase-8 were not significantly altered after 14 days following exposure to an EMF. These results indicate that cardiac tissues are physiologically well buffered against the negative effects of electromagnetic stress and can maintain cellular processes and functions when exposed to an EMF. Nevertheless, upon the addition of even a weak co-stimulator (under hypoxic or oxidative stress conditions), tolerable EMF exposure might induce impairments in mitochondrial metabolism and apoptosis in the brain, liver, and renal tissue in rats [46,47]. This response may be due mostly to the increase in cellular ROS levels and defects in the antioxidant system mediated by EMF exposure [4]. In our study, the myocardial expression levels of *SOD2* and *catalase*, the first line of the ROS-scavenging systems, were examined to further evaluate the effects of EMF exposure on cardiac antioxidant capacity under electromagnetic stress. Analysis of the expression levels of *SOD2* and *catalase* genes demonstrated that myocardial antioxidant defense remained constant in the EMF-challenged mice. These data regarding antioxidant capacity in relation to metabolic and apoptotic cell statuses are in line with many previous studies on cell and animal models, which demonstrated the absence of marked effects on the antioxidant system under conditions of electromagnetic stress [48,49,50]. However, several studies have reported that 900 MHz EMF exposure may cause transcriptional changes in oxidative stress status and apoptosis-related genes in the brain [2] and in cancer cells [51]. These contradictory data may be due to the use of different cell models, experimental protocols, and durations of EMF exposure. Indeed, we have recently demonstrated that at the metabolic level, long-term exposure to EMFs could affect mitochondrial oxidative machinery through modulating cardiac OXPHOS capacity in a mouse model [52]. The study indicated that exposure to a 915 EMF for 28 days promotes the cardiac respiratory capacity of mitochondria without compromising the structural integrity of the heart tissues in mice at the age of 9 months. These results suggest an important role of OXPHOS-dependent metabolic reprogramming in cardiac adaptation under prolonged electromagnetic stress. In the current study, we demonstrated that 915 MHz radiofrequency exposure for 14 days does not induce mitochondrial OXPHOS defects in 8-week-old mice, indicating a state of cellular energy balance in the EMF-challenged hearts. Thus, the discrepancies between the two studies may be due to the different ages of the mice and durations of the EMF exposure protocols. 

Alterations in inflammatory status are among the sensitive parameters that can be used to elucidate the cellular stress response to electromagnetic stress. The short- and long-term exposure to an EMF can compromise the integrity of the immune system as it can damage immune cells and irreversibly affect some immune functions [14]. In our experimental protocol, no significant changes in the structural integrity of the cardiac tissue of EMF-exposed mice were found. In addition, the results from the analysis of fibro-inflammatory cytokines including *TGF β-1*, *IL-6*, and *CCL2* genes showed unchanged myocardial fibrotic and inflammatory statuses of the myocardium after exposure to an EMF for 14 days. Interestingly, a recent study suggested that a 900 MHz EMF can activate the brain and kidney renin–angiotensin system, and this activation may be related to inflammatory reactions [53]. In addition to cardiac inflammatory status, in the present study, we evaluated myocardial collagen content and the expression of fibrosis-related genes in the EMF-exposed mice. Importantly, in our experimental protocol, the total collagen content and gene expression of the predominant components of cardiac ECM *collagen types I* and *III* were comparable between the control and EMF-challenged hearts, suggesting that EMF exposure for 14 days does not induce fibrotic tissue remodeling. There is a lack of consistent results concerning the remodeling processes in cardiac tissue of human origin. Therefore, future studies should also address the integrative analysis of the biological effects of EMFs on different tissues and organs in humans. 

## 4. Materials and Methods

### 4.1. Animals

This investigation conformed to the Guide for the Care and Use of Laboratory Animals published by the US National Institutes of Health (NIH Publication No. 85-23, revised 1985) and was performed in accordance with the recommendations of the French Accreditation of the Laboratory Animal Care (approved by the local ethics committee). Eight-week-old Swiss Webster outbred male mice (Envigo RMS, Gannat, France) were used for the in vivo experiments. Mice were housed in groups of four in polycarbonate cages and were allowed free access to standard food and water. Temperature was controlled at 21 ± 2 °C. Two days before EMF exposure, mice were allowed to adapt to new environmental conditions (light was provided on a 12 h light–12 h dark cycle), as previously described [52]. Animals were randomly divided into two groups: the control sham (*n* = 10) and EMF (*n* = 10) groups. The mice from EMF group were exposed to a 915 MHz EMF for 14 days (9 h per day). The control group was not exposed to an EMF. Animals were sacrificed, and hearts were removed and rinsed in 4 °C phosphate buffer saline (PBS).

### 4.2. Radiofrequency Equipements

**Figure 7:**
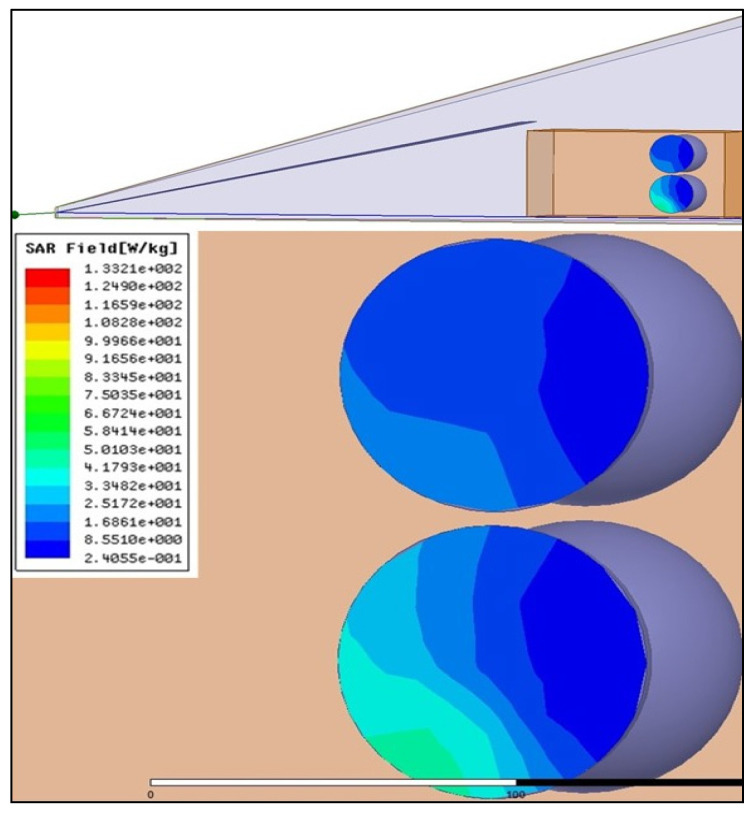
Electromagnetic computation of SAR of six phantoms in GTEM. The impact of EMF on the mouse body was calculated using a standardized unit known as the SAR. The electromagnetic simulations for the six phantoms were conducted using HFSS software. The whole-body SAR was determined as the volume integration of absorption power using MATLAB codes.

In vivo protocol was conducted using a Giga-TEM (GTEM) cell, in which the animals were subjected to EMF. The 915 MHz band was selected as the EMF level to which the mice would be exposed. It is a commonly used band in the Global System for Mobile Communications (GSM) and accounts for a large share of the total electromagnetic energy emitted to the environment by mobile systems. A solid-state radiofrequency generator with a fixed frequency of 915 MHz (WSPS-915–1000) (Chengdu Wattsine Electronics Technology, Chengdu, Sichuan, China) was used for the experiments. The interactions of EMF with animal models were defined in terms of specific absorption rate (SAR). In this study, the SAR was determined numerically and expressed in units of watts per kilogram (W/kg), as previously described [54]. Electromagnetic computations were performed using High Frequency Structure Simulator (HFSS) software, version 11.1. The GTEM was powered using 1 W of input power at 915 MHz. In this configuration, six phantoms were placed in two rows perpendicular to the GTEM axis (Figure 7). The variation of the SAR in the plane of symmetry shows that the maximum value approaches 50 W/kg, corresponding to 200 W/kg when a 4 W generator is used in real conditions. In a mouse model, the average value of the SAR calculated on the basis of the extracted data was ~40 W/kg.

### 4.3. Western Blot

Extraction of proteins from cardiac tissues was performed as previously described [52]. Briefly, we used RIPA buffer for protein extraction, and lysates were quantified after clarification using the Bio-Rad Protein Assay (Bio-Rad, Hercules, CA, USA). Proteins were resolved via SDS-PAGE and Western blotting. Immunoreactive bands were detected via chemiluminescence using the Clarity Western ECL Substrate (Bio-Rad, Hercules, CA, USA) on a ChemiDoc MP Acquisition system (Bio-Rad, Hercules, CA, USA). Antibodies used in this study were MitoProfile^®^ Total OXPHOS Rodent WB Antibody Cocktail, SOD2, caspase-8, BAX, BCL-2, and β-Actin from Santa Cruz Biotechnology (Santa Cruz, CA, USA). β-Actin was used as a loading control.

### 4.4. Collagen Content Detection

Picrosirius red staining of cardiac sections was performed for the histological visualization of collagen fibers (fibrosis). The evaluation of cardiac fibrosis was quantified using ImageJ (Fiji, version 2.0.0-rc-69/1.52; RRID:SCR_003070) as previously described [52].

### 4.5. Quantitative RT–PCR Analysis

The expression of genes was assessed using qRT-PCR. Total RNAs were isolated from cardiac tissue using the RNeasy mini kit (Qiagen, Hilden, Germany) and reverse-transcribed as previously described [52]. Primer sequences are detailed in Table 1. The expression of target gene was normalized to HPRT expression.

### 4.6. Statistical Analysis

Data are presented as means ± SEM. Statistical analysis between two groups was performed via Student’s *t*-test using GraphPad Prism (version 5.0; GraphPad software), *p*-values < 0.05 were considered statistically significant.

## 5. Conclusions

In conclusion, our findings suggest that 915 MHz radiofrequency exposure for 14 days does not induce significant alterations in the apoptosis, metabolism, and antioxidant statuses of the myocardium in mice. In addition, no exposure-related effects were noted in relation to fibro-inflammatory markers and heart tissue integrity. Further studies exploring alternative and commonly used frequencies with longer exposure times are needed to conclusively determine the impact of EMF on the cardiovascular system. 

## Figures and Tables

**Table 1:** Real-time qPCR primer sequences.

Gene	Forward Sequence (5′-3′)	Reverse Sequence (5′-3′)
*BAX*	GGCGAATTGGAGATGAACTG	CCCCAGTTGAAGTTGCCAT
*BCL2*	GATGACTGAGTACCTGAACCG	CAGAGACAGCCAGGAGAAATC
*Catalase*	CGAAGGTGAAGCAGGACATG	CTCCAGTAGCCAAAGATCAAGG
*CCL2*	GTCCCTGTCATGCTTCTGG	GCTCTCCAGCCTACTCATTG
*Collagen type I*	TGTGTGCGATGACGTGCAAT	GGGTCCCTCGACTCCTACA
*Collagen type III*	AAGGCGAATTCAAGGCTGAA	TGTGTTTAGTACAGCCATCCTCTAGAA
*HPRT*	TGAAAGACTTGCTCGAGATGTCAT	TCCAGCAGGTCAGCAAAGAA
*IL-6*	CAAAGCCAGAGTCCTTCAGAG	GTCCTTAGCCACTCCTTCTG
*SOD2*	GGACAAACCTGAGCCCTAAG	CAAAAGACCCAAAGTCACGC
*TGF β-1*	GAGCCCGAAGCGGACTACTA	CACTGCTTCCCGAATGTCTGA

## Data Availability

The raw data supporting the conclusions of this article will be made available by the authors without undue reservation.
